# Cancer awareness through school curriculum: evidence and recommendations from a quasi-experimental study among school children in the State of Kerala, India

**DOI:** 10.3332/ecancer.2025.1876

**Published:** 2025-03-20

**Authors:** Phinse Mappalakayil Philip, Neethu Ambali Parambil, Maya Padmanabhan, Satheesan Balasubramanian

**Affiliations:** 1 Department of Preventive and Community Oncology, Malabar Cancer Centre - Post Graduate Institute of Oncological Sciences and Research, Thalassery 670103, Kerala, India; 2 Department of Clinical Research and Biostatistics, Malabar Cancer Centre - Post Graduate Institute of Oncological Sciences and Research, Thalassery 670103, Kerala, India; 3 Malabar Cancer Centre - Post Graduate Institute of Oncological Sciences and Research, Thalassery 670103, Kerala, India; a https://orcid.org/0000-0002-6719-0641

**Keywords:** school curriculum, cancer prevention, cancer awareness, cancer education

## Abstract

**Introduction:**

Cancer is emerging as a leading cause of morbidity and mortality in India and other countries worldwide. Preventable risk factors for cancer, such as the use of tobacco and alcohol, unhealthy diets and physical inactivity, are often initiated and established during adolescence. Identifying effective strategies for engaging adolescents in cancer-protective behaviours is essential. The current study investigates the potential of schools as settings and school curricula as tools for raising cancer awareness among teenagers.

**Methods:**

Consultative meetings and workshops were conducted with education, health and social services experts to develop a primer for cancer control in the school curriculum. Textbooks were scanned to determine the extent of cancer-related topics for standards five to ten. The study participants’ awareness at baseline was assessed using the Cancer Awareness Measure toolkit (version 2.1). Based on these findings, a study package was developed and implemented through trained teachers. A quasi-experimental study design was used to assess the impact of the study package in improving cancer awareness.

**Results:**

In the post-intervention survey, the participants from the intervention schools demonstrated significant improvements in their understanding of cancer warning signs (unexplained lump or swelling (*p* = 0.0001), unexplained bleeding (*p* = 0.007), persistent cough or hoarseness (*p* = 0.0001), non-healing ulcers (*p* = 0.0001) and risk factors (consumption of fewer quantities of fruit and vegetables (*p* = 0.010), eating red or processed meat (*p* = 0.0001) and physical inactivity (*p* = 0.01)).

**Conclusion:**

Implementing the cancer awareness study package through classroom teaching improved students’ understanding of warning signs and risk factors for cancer. The study emphasised the role of the school as a setting, the school curriculum as a tool and teachers as promoters of cancer prevention education. However, there is no guarantee that this program will work if the learners’ environment is not improved through the concurrent teaching of parents. Children learn what they live.

## Introduction

The incidence of cancer worldwide is expected to increase to 30.2 million by 2040, which is a 57% increase from 2020 levels [1]. The World Health Organization (WHO) estimates that nearly 30% to 50% of all cancers can be prevented through primary prevention strategies such as diet and lifestyle modifications, avoidance of tobacco and alcohol and vaccination against certain viruses [2]. Generally, carcinogenesis involves a series of genetic alterations in normal cells over a long period [3]. A basic understanding of these risk factors and appropriate changes in lifestyle and diet can aid in the primary prevention of cancer. Secondary prevention strategies such as screening and early diagnosis improve survival outcomes in patients with cancer. Awareness of warning symptoms of cancer promotes health-seeking and early diagnosis [4]. Adolescents and young adults should be aware of the risk factors for and warning signs of common cancers. Preventable risk factors for cancer, such as tobacco and alcohol use, unhealthy diets and physical inactivity, are often initiated and established during adolescence [5]. Youths can be prevented from developing cancer in the future by making changes to their lifestyles and behaviours.

Various studies have reported a high prevalence of behavioural and lifestyle risk factors among adolescents [6, 7]. The Global Youth Tobacco Survey revealed that nearly one-tenth of school children aged 13–15 currently use tobacco in some form in India [6]. The survey further reported that only 37.8% of learners were taught about the ill effects of tobacco use in their classes [6]. The WHO reported that 18% of children between the ages of 5 and 19 were either obese or overweight. Obesity increases the risk of certain cancers [7]. More than 80% of adolescents do not perform sufficient physical activity. Regular physical activity helps prevent several cancers and other non-communicable diseases [8]. Adolescents do not understand important warning signs of cancer [5]. A study from Australia reported poor awareness of cancer risk factors and symptoms among adolescents [9].

School health education can potentially improve public health [10]. Curriculum-based studies, such as the St. Jude Cancer Education for Children Programme (USA) (SJCECP), help increase cancer awareness in school children [11]. The ‘Changing the Course’ curriculum is an innovative school-based cancer control program that provides children with the knowledge, skills and attitudes necessary to adopt cancer risk-reducing dietary practices [12]. A study from South India on the need for a school-based cancer education program also revealed that adding cancer-related themes to the curriculum will increase awareness [13]. The identified models in school-based cancer education, such as the ‘Changing the Course’ [12] curriculum and the SJCECP [14], developed a curriculum for cancer awareness among students. These intervention studies did not explore the potential of existing school curricula in cancer awareness by systematically incorporating such messages in regular classroom lessons. The current study investigated the potential for introducing cancer-related information to the existing school curriculum to improve cancer awareness among school children.

## Methodology

The study was part of a larger initiative by the Government of the State of Kerala, India, to achieve sustainable development goals. Sustainable Developmental Goal number three, target 3.4 adopted by United Nations member states in 2015, encourages all member states to ‘reduce by one-third premature mortality from noncommunicable diseases by 2030 through prevention and treatment and promote mental health and well-being’ [15]. In this context, the Government of Kerala instructed the Comprehensive Cancer Centre in northern Kerala to develop a primer for cancer control to include the same primer in the school curriculum. The Comprehensive Cancer Centre undertook extensive conversations with the District Institute for Education and Training (DIET) about curriculum development initiatives to incorporate cancer control and decided to continue the consultative process with experts from related fields. Two workshops were conducted with experts from the fields of education, school curriculum development, healthcare, social service, law enforcement and media. Student representatives also participated. The workshop report presented the following recommendations.

1. The current school syllabus is scanned to explore the breadth and nature of the cancer-related themes already present.
2. Surveys among students to determine their current level of cancer awareness.
3. Prepare a study package for cancer health education based on textbook scanning and baseline survey results.
4. To conduct appropriate scientific research to assess the benefits and feasibility of the curriculum-based ‘study package’ in improving cancer awareness among school children.

### Study design

A quasi-experimental study design was used for conducting the study. In this experimental study, participants in the intervention and control groups were non-randomly selected. Participants in the control group received the intervention after completing the final data collection. All the 9th-standard students from four public schools in an educational sub-district in northern Kerala were included in the study. The learners from the 9th standard were selected for the study due to practical considerations concerning the implementation of the program. Two schools were located in rural areas, and the other two were in urban areas. The intervention and control groups included one school from an urban or rural area. A total of 627 students participated in the study, 319 of whom were in the intervention group. Before the initiation of the study, a parent-teacher association meeting was held at all the participating schools, and the study objectives and methodology were explained.

### Data collection tool

The Cancer Awareness Measures (CAM) toolkit version 2.1, which was developed and validated by Cancer Research UK [16], was utilised for the baseline and final surveys. The CAM questionnaire was translated to the regional language Malayalam and back-translated to English until a conceptually equivalent version of the questionnaire was obtained in Malayalam. The questionnaire in the regional language was further evaluated in a workshop by experts from the DIET and MCC to assess the appropriateness of language for use among adolescent children. Questions found to be irrelevant to the local setting in which the study was conducted were excluded, and relevant changes were made to the selected questions. The questionnaire was further piloted among school children to assess its face validity. The study protocol received approval from the Institutional Review Board and Institutional Ethics Committee (1617/IRB-IEC/13/MCC/25-4-2018/5 dated 25th May 2018).

### Textbook scanning

Textbooks of all the subjects from class 5 to class 10 were examined by an expert panel from DIET to identify any mention of anything relevant to cancer awareness in the existing school curriculum. They also identified areas that can be utilised for incorporating awareness-related messages. This was done in two phases. A preliminary general evaluation was followed by detailed subject-wise exploration and listing.

### Baseline survey and evaluation

Cancer-related awareness of the study participants was assessed via the CAM questionnaire before the intervention. Data collection was performed under the supervision of faculties from DIET. The baseline survey findings guided the development of the study package.

### Development of the study package

Based on the findings of the textbook scanning and baseline survey, a study package was developed for intervention in the schools. A 2-day workshop was conducted to develop the study package. In addition to the DIET and MCC faculty members, high school teachers also participated in the workshop. Four subjects were selected to assess cancer awareness among school children. Chapters were identified from the Social Science, Biology, Chemistry and Malayalam textbooks of the 9th standard for incorporating the relevant cancer control messages. An 18-page booklet was prepared in the workshop to aid the teachers in delivering the study package.

### Training for teachers

Teachers who taught social science, biology, chemistry and Malayalam subjects in the intervention and control group schools were given 1-day training for implementing the cancer awareness study package.

### Study package implementation at intervention schools

The study package was implemented in the intervention schools over 4 months through trained teachers during regular class hours. They were incorporated into the regular curriculum along with the relevant topics of the subjects mentioned.

### Continuous evaluation and monitoring of the program

The implementation of the study package was monitored via DIET by visiting intervention schools during class hours. Meetings were conducted with school headmasters and teachers to review the progress and identify the hurdles faced by the teachers in delivering awareness messages through classroom teaching.

### Final survey and evaluation

The level of cancer awareness of the study participants in the intervention and control groups was assessed via the same CAM questionnaire 2 months after the implementation of the study package. The study package was implemented in the schools in the control group after the final survey.

### Evaluation and primer preparation

**Figure 1.**
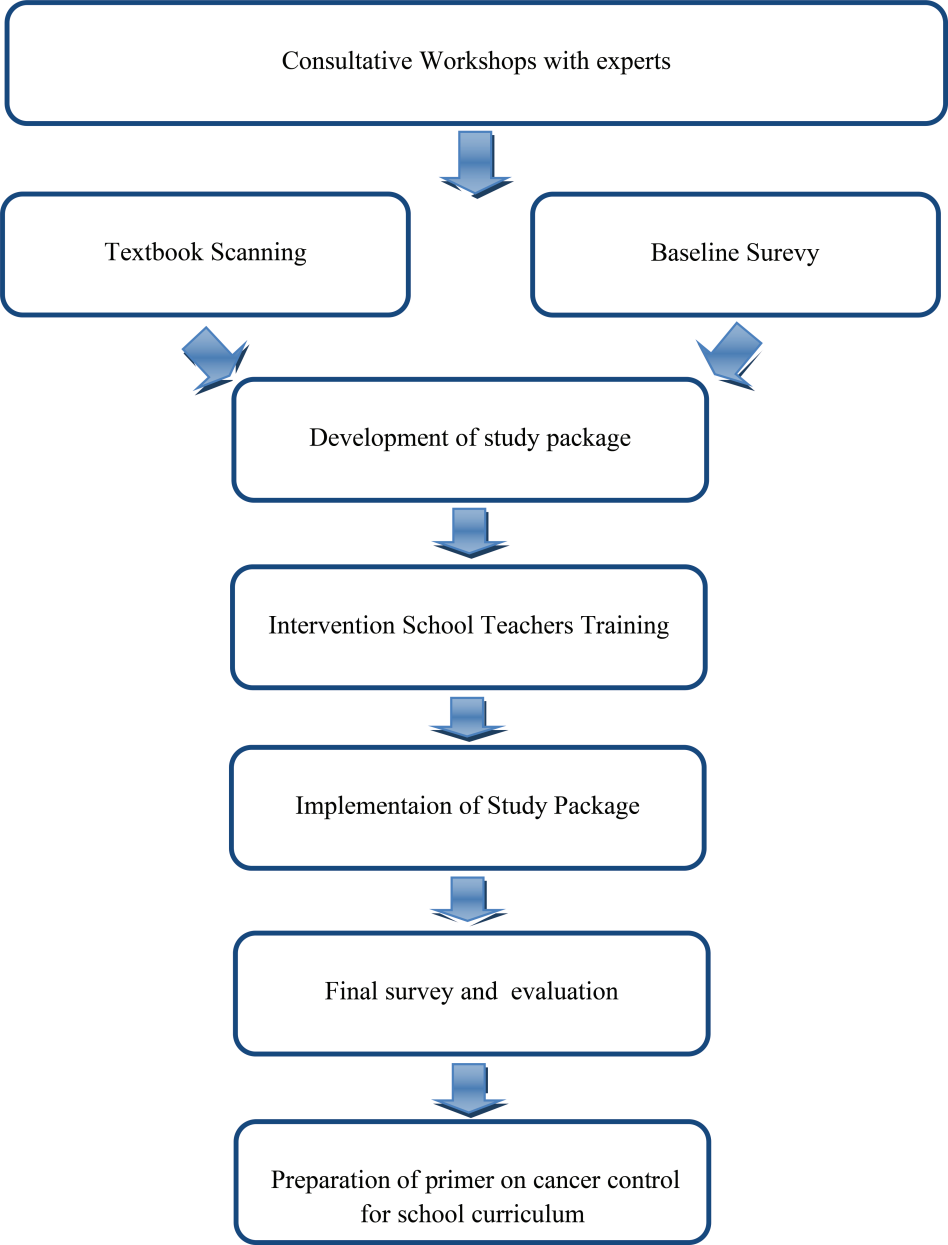
Study flowchart.

The study results were evaluated in a series of post-evaluation meetings and the final report with findings and recommendations was prepared and submitted to the government for consideration during the next curriculum update. The final report consisted of a primer on cancer-related topics to be included in the school curriculum in various classes (Figure 1).

## Results

### Textbook scanning

Textbooks of all the subjects from classes five to ten were manually searched by a team of teachers from the DIET, and the mention of cancer-related topics was limited. Twenty-nine relevant areas from various subjects were identified, which contained certain ideas and threads that, if developed, could provide insights into cancer control, even though no explicit mention of cancer or cancer control was present. A team of doctors from the Comprehensive Cancer Care Centre assessed the areas identified for their potential for incorporating relevant cancer control messages and suggested a set of topics appropriate for each identified area (Table 1).

### Awareness about cancer warning signs and symptoms

This study reported statistically significant improvements in awareness of the following cancer symptoms among the intervention group compared with the control group participants in the post-intervention survey. They include ‘Unexplained lump or swelling’, ‘Unexplained bleeding’, ‘Persistent cough or hoarseness’ and ‘A sore that does not heal’ (Table 2). The study did not yield a significant improvement in awareness of other symptoms, such as ‘persistent unexplained pain’, ‘persistent change in bowel/bladder habits’, ‘persistent difficulty swallowing’ and ‘unexplained weight loss’.

### Awareness of cancer risk factors

Statistically significant improvements in awareness of cancer risk factors, such as ‘low intake of fruits and vegetables’ (*p* = 0.01), ‘consumption of red meat’ (*p* = 0.001) and ‘absence of regular exercise’ (*p* = 0.01), were observed. Awareness of risk factors such as ‘obesity’ and ‘old age’ did not significantly improve through the intervention. The baseline survey revealed high awareness of risk factors such as tobacco smoking and alcohol use in both the intervention and control groups. Despite this, no significant improvements in awareness of tobacco (pretest (77.74%), posttest (88%)) and alcohol (pretest (72%), posttest (81.4%)) were observed in the intervention group after the implementation of the study package.

### Awareness of early detection of cancer

In the intervention group, awareness of the feasibility of early detection of oral cavity cancers (*p* = 0.008), breast cancers (*p* = 0. 0001) and the incidence of cervical cancer (*p* = 0.065) improved compared with that in the control group in the post-study survey.

## Discussion

To the best of our knowledge, this is the first study to evaluate the feasibility and effectiveness of a school curriculum-based cancer awareness intervention. The school curriculum-based intervention resulted in an improvement in general awareness of cancer symptoms and risk factors. Furthermore, the study also demonstrated the feasibility of implementing the intervention as part of the regular school curriculum by training the teaching faculty at the school. Few studies from developed countries have demonstrated that school settings can impart cancer awareness with tailored curricula as tools [12, 14] and teachers as resource persons [17]. The potential of school as a setting and teachers as promoters of cancer prevention was piloted in a program conducted in the United States. It was showcased as a validation of the strategy to use no healthcare professionals to impart cancer awareness among the selected population [17]. Our study findings further reinforce the role of teachers and school settings in cancer prevention interventions.

The study revealed that the existing school curriculum lacks explicit messages on cancer prevention. Even though the major modifiable risk factors for cancer, such as tobacco use, alcohol use, unhealthy food and lifestyle, begin in adolescence, the absence of cancer prevention-related health messages in school textbooks is astonishing. The curriculum development committees failed to recognise the potential of the school curriculum as a tool for the primary prevention of cancer. This was evident from the findings of the textbook scanning conducted in the present study and warrants the immediate attention of policymakers. The study explored the possibility of providing learning experiences to create cancer awareness in the existing school curriculum and identified a few areas in different textbooks from various classes that have a scope for incorporating cancer awareness messages. To capitalise on these opportunities, the study package developed as part of the intervention linked these areas to various types of cancer-related information. For example, the Chemistry chapter on ‘Elements and the periodic table’ was linked to heavy metals, which are carcinogens. Similarly, the chapter on ‘nutrition and exercise’ was connected to the ill effects of obesity and resulting lifestyle diseases, including cancer, through the package.

The poor awareness of cancer signs, symptoms and risk factors observed in the baseline survey suggests that schools and communities failed to impart adequate cancer-related knowledge to school children. Several studies from across the world have reported poor cancer awareness among adolescents [4, 9, 18]. Adolescence is an ideal period in the life of a person to initiate responsible health behaviours. Unfortunately, most risky lifestyle behaviours are initiated during adolescence[19] and these behaviours are predictors of future lifestyle diseases, including cancer. Adopting a healthy lifestyle during adolescence lowers the risk of having cancer later in life [20].

The intervention package developed and implemented in the present study was embedded in the existing school education system. The intervention aimed to improve the awareness of participants in warning signs of cancer, causes of cancer and early detection of cancer. This curriculum-based intervention resulted in a greater uptake of cancer messages among the participants. This was especially true for warning signs such as an unexplained lump or swelling, unexplained bleeding, persistent cough and nonhealing ulcers. A school-based educational intervention study conducted in the UK reported a statistically significant improvement in cancer symptom awareness among adolescents after the intervention [18].

The intervention also improved participants’ awareness of risk factors for cancer, namely, low intake of fruits and vegetables, use of red meat and absence of physical activity. Generally, adolescents are uninformed about the role of diet and lifestyle choices and the risk of developing cancer [21]. The majority of the study participants in both the intervention and control groups were aware of the risk of cancer due to smoking, passive smoking and alcohol use. A study from Kerala reported high awareness of the harmful effects of tobacco among high school and higher secondary school students [22]. This is also a reflection of the successful implementation of tobacco control policies in the state of Kerala [23].

The curriculum-based intervention implemented in the study did not improve awareness of the association between being overweight and the risk of developing cancer. There is a need to inculcate in children that being overweight increases the risk of cancer and regular exercise can help reduce the risk of cancer. A few warning signs, such as changes in bowel and bladder habits and persistent difficulty swallowing, were not recognised as probable cancer symptoms by the participants even after the completion of the intervention. We consider these shortcomings and limitations of our intervention.

To increase awareness related to cancer among children, the school curriculum should include learning ideas, activities and experiences related to various subjects. In the first phase, learning experiences should be arranged in a hierarchical order according to age in various topics from grades 5 to 10. The topics recommended for inclusion in the school curriculum for cancer control should be included in the textbook as simple units or lessons (Table 3). Teachers should be given the necessary training to impart cancer awareness as part of regular classroom teaching.

## Conclusion

School curriculum-based educational intervention is a novel strategy for cancer awareness among adolescents. The curriculum-based intervention resulted in significant improvement in participants’ understanding of the causes, symptoms and early detection of cancer. Policymakers should consider school curricula as useful tools for cancer prevention, as most of the preventable risk factors for cancer are often initiated and established during adolescence. The intervention model can be implemented in all schools in the state with no additional manpower or resource requirements once the necessary modifications are incorporated into the school curriculum and teacher training programs. We can conclude that a school as a setting and a school curriculum as a tool facilitate the generation of cancer awareness among adolescents. However, we should also consider the importance of an enabling environment in school and community that favors the adoption of the cancer prevention strategies outlined in the curriculum. To the best of our knowledge, this is the first study that evaluated regular school textbooks for possible cancer-related content and transformed them into a vehicle for cancer awareness.

## Conflicts of interest

All authors report no relationships that could be construed as a conflicts of interest.

## Funding

The authors state that no external funding was received for this study. In addition, the authors certify that this work has no financial conflicts of interest.

## Author contributions

The authors confirm their contribution to the paper as follows. Study conception and design: SB, PMP, NAP, data collection PMP, NAP; analysis and interpretation of results: MP, SB, PMP, NAP, draft manuscript preparation PMP, NAP. All authors reviewed the results and approved the final version of the manuscript.

## Figures and Tables

**Table 1.** List of subject areas identified from existing school textbooks for incorporating cancer control messages.

S/N	Class	Subject	Identified areas with potential for incorporating cancer control messages	Suggested cancer control topics corresponding to the area identified
1	5th standard	Malayalam (Language)	Discussion on agriculture	Eat fruits & vegetables Do physical activity
2	5th standard	Malayalam (Language)	Story on cancer-related experience by a film actor	Reduce fear and stigma Scientific treatment Cancer is curable
3	5th standard	Hindi (Language)	Comparison of rural-urban life, industrialization	Outdoor air pollution Cancer risk factors
4	5th standard	Hindi (Language)	Bottled water and beverages	Unhealthy food habit Food adulteration
5	5th standard	General Science	Disease prevention	Cancer prevention
6	5th standard	Social science	Dependence on other states for rice and vegetables	Eat fruits & vegetables Do physical activity
7	5th standard	Social science	Discussion on the family budget	Treatment expenses Health insurance
8	5th standard	Hindi (Language)	Story of stephen hopkins, Oscar Pistorius	Cancer is curable Cancer survival Rehabilitation
9	6th standard	Hindi (Language)	Importance of agriculture	Eat fruits & vegetables
10	6th standard	General Science	Food habits	Overweight and obesity Diet
11	7th standard	Social science	Climate change	Air pollution
12	8th standard	General science	Food	Overweight and obesity Diet
13	8th standard	Hindi	Letter to doctor	Cancer prevention Lifestyle and diet Physical activity
14	8th standard	Hindi	Love for sweets	Unhealthy diet
15	8th standard	Chemistry	Atom and elements	Radiation Radiotherapy Heavy metals
16	9th standard	English	Environmental destruction due to construction	Environmental pollution
17	9th standard	Hindi	Adopting new food habits	Diet and life style
18	9th standard	Hindi	Lack of safe drinking water	Safe food and water
19	9th standard	Hindi	A story about a handicapped person	Cancer rehabilitation Cancer survivorship
20	9th standard	Chemistry	Isotopes	Cancer treatment
21	9th standard	Chemistry	Ozone layer depletion	Ultraviolet rays and skin cancer
22	10th standard	Hindi	Drinking water	Safe food and water
23	10th standard	Hindi	Herbal medicine	Scientific treatment
24	10th standard	Chemistry	Periodic tables Radioactive elements	Radiation Radiotherapy
25	10th standard	Chemistry	Isotopes	Cancer treatment Nuclear medicine
26	10th standard	Chemistry	Alcohol Benzene	Alcohol &cancer Carcinogens
27	10th standard	Chemistry	Green chemistry Chemistry in daily life	Health & environment
28	10th standard	English	Leading bad life: alcohol and gambling	Alcohol and cancer
29	10th standard	English	Father used to smoke	Tobacco and cancer Second hand smoke

**Table 2.** Number of participants who correctly identified warning signs and symptoms of cancer.

S/N	Sign/symptom	Intervention Baseline (*n* = 266)	Control Baseline (*n* = 262)	*p* value	Intervention Final (*n* = 301)	Control Final (*n* = 281)	*p* value
1	Unexplained lump or swelling	63	66	0.867	184	106	0.000
2	Unexplained Bleeding	61	77	0.206	136	98	0.007
3	Persistent cough or hoarseness	109	108	0.76	204	129	0.000
4	Change in appearance of a mole	32	31	0.506	63	41	0.086
5	A sore that does not heal	44	63	0.002	163	82	0.000

**Table 3.** Recommended topics for inclusion in the school curriculum for cancer control.

S/N	Class	Recommended topics
**1**	5th standard	The concept is that cancer can be cured if detected early. Cancer is not something to be terrified of. (Autobiography of cancer patients) Information on factors that increase the risk of cancer like smoking, passive smoking, alcohol consumption, changes in food habits, and a lack of exercise. The importance of healthy eating habits and exercise should be highlighted.
**2**	6th standard	Extension of ideas from class 5 Certain cancers can be discovered early and cured completely. Autobiography of cancer survivors Avoiding alcohol and smoking can help prevent cancer.
**3**	7th standard	If you observe any abnormal changes in your body, seek medical attention. The psychological effects of cancer. Society's approach to cancer (examples may be provided). Changing bad eating habits like avoiding fast food and junk food. The importance of including fruits and vegetables in our regular diet. Cancer prevention measures. Eliminating the habit of using drugs.
4	8th and 9th standard	What is cancer Different types of cancers Symptoms of cancer Changes in the body and consequences Scientific treatment Society's perspectives on cancer Treatment schemes, schemes and treatment centers Estimation Risk factors – being overweight, red meat, etc. Age and cancer Cancer and gender differences Early detection and testing method Preventive measures
**5**	10th standard	What is cancer (biological) Common cancer symptoms Different types of cancers Cancer risk factors Scientific cancer treatment Early detection, self-examination Financial security and treatment options Responsibility for social cohesion Common cancers Screening tests Cancer prevention strategies Cancer myths and facts Shaping the public perception of cancer.
